# NAChRDB: A Web Resource of Structure–Function Annotations to Unravel the Allostery of Nicotinic Acetylcholine Receptors

**DOI:** 10.1021/acsomega.1c00817

**Published:** 2021-08-31

**Authors:** Aliaksei Chareshneu, Purbaj Pant, Ravi José Tristão Ramos, David Sehnal, Tuğrul Gökbel, Crina-Maria Ionescu, Jaroslav Koča

**Affiliations:** † CEITEC - Central European Institute of Technology, Masaryk University, Brno 601 77, Czech Republic; ‡ National Centre for Biomolecular Research, Faculty of Science, Masaryk University, Brno 625 00, Czech Republic; § Protein Data Bank in Europe (PDBe), European Molecular Biology Laboratory, European Bioinformatics Institute (EMBL-EBI), Wellcome Genome Campus, Hinxton, Cambridge CB10 1SD, U.K.; ∥ Department of Molecular Biology and Genetics, Izmir Institute of Technology, İzmir 35430, Turkey

## Abstract

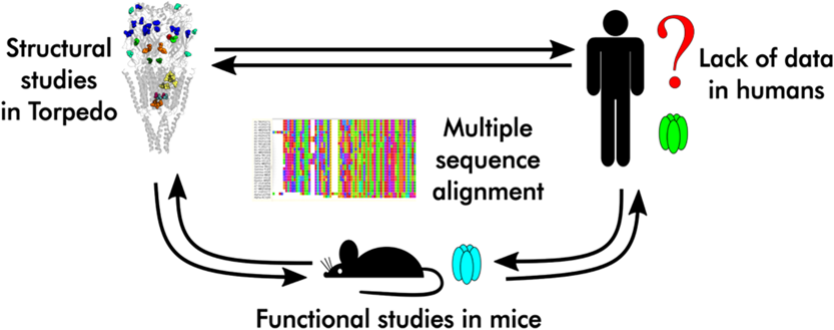

Nicotinic acetylcholine receptors (nAChRs) comprise a large and ancient family of allosteric ion channels mediating synaptic transmission. The vast knowledge about nAChRs has become difficult to navigate. NAChRDB is a web-accessible resource of curated residue-level functional annotations of neuromuscular nAChRs. Interactive three-dimensional (3D) visualization and sequence alignment give further context to this rich and growing collection of experimental observations and computational predictions. NAChRDB is freely available at https://crocodile.ncbr.muni.cz/Apps/NAChRDB/, with interactive tutorials and regular updates to the content and web interface. No installation or user registration is required. NAChRDB is accessible through any modern internet browser on desktops and mobile devices. By providing immediate and systematic access to practical knowledge gained through decades of research, NAChRDB represents a powerful educational tool and helps guide discovery by revealing gaps in current knowledge and aiding the interpretation of results of molecular and structural biology experiments or computational studies.

## Introduction

Nicotinic acetylcholine receptors (nAChRs) mediate synaptic transmission by converting the chemical signal of acetylcholine into ion current across the cell membrane. This function requires complex communication between the agonist-binding pocket in the extracellular domain and a gating mechanism lying 60 Å away in a channel traversing the entire protein.^1^ NAChRs contribute to many physiological and pathological processes, from central nervous system diseases^2^ to COVID-19 infection,^3^ and are targeted by hundreds of compounds whose pharmacological action relies on allosteric modulation of channel gating.^4,5^

Not surprisingly, nAChRs have remained the focus of intensive research for more than 50 years.^6^ This immense effort has produced thousands of experimental observations and computational predictions for different receptor types, using inconsistent residue numbering schemes and terminology. Furthermore, because nAChRs are large and difficult to crystalize, structural studies have focused on individual parts of the molecule and neglected to account for the allosteric aspect, which is critical for nAChR function. In the absence of comprehensive and unified structural annotation, it is almost impossible to identify gaps in knowledge or areas of controversy and to promote further discoveries.^7,8^ NAChRDB addresses such limitations by providing web-based, systematic access to curated residue-level functional annotations of nAChRs, with interactive three-dimensional (3D) visualization and sequence alignment.

## Methods

**Figure 1:**
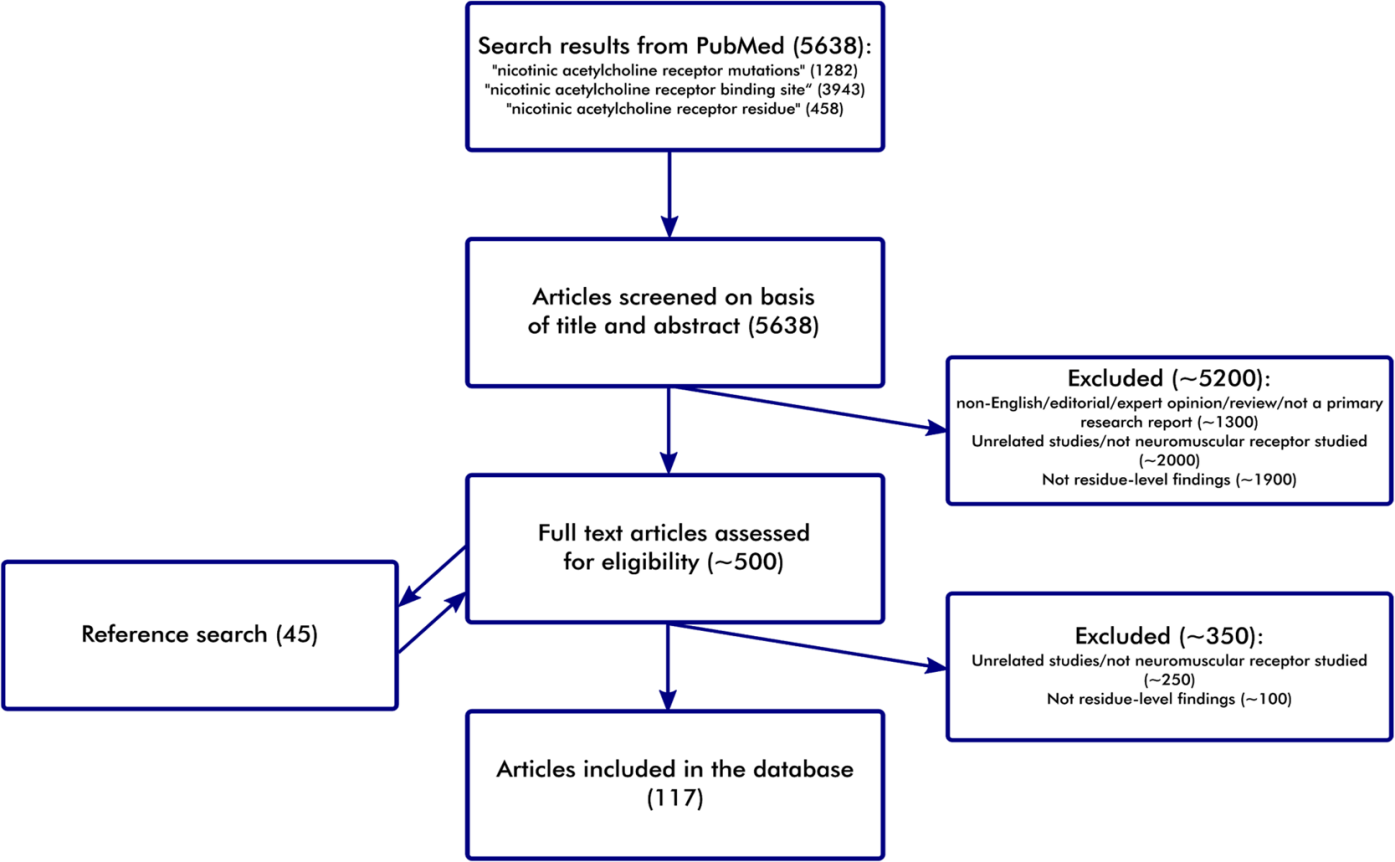
Flowchart of the literature scanning process. Functional annotations were collected from a semisystematic literature review of Medline through PubMed. First, we searched PubMed for articles describing the functional role of neuromuscular nicotinic acetylcholine receptor (nAChR) amino acid residues. Next, we briefly reviewed the titles and abstracts and excluded unsuitable papers. We then reviewed the full text of the remaining articles and retained only relevant papers that covered studies using subunit types, chain IDs, and residue numbering corresponding to the canonical sequences of nAChR subunits.

The models of complete nAChR structures were obtained from the Protein Data Bank, whereas the sequences of complete nAChR subunits were obtained from UniProt. Functional annotations were collected from a semisystematic literature review of Medline through PubMed (Figure 1). All original annotations were curated manually and mapped onto homologous residues using sequence alignment. Additionally, we conducted two computational studies to complement current knowledge. Residues potentially involved in charge transfer networks facilitating channel gating were predicted using a modified charge-profile analysis.^9^ Channel-lining residues were predicted using ChannelsDB.^10^ All predictions were added to NAChRDB as annotation records, with full reference to the source of information. Full details are available in the Supporting Information Methods.

## Results and Discussion

### Database Coverage

NAChRDB currently contains approximately 2000 unique annotation records describing the functional role of specific residues, as inferred based on experimental observations or computational predictions reported for nAChRs of species from six genera. These data come from 117 studies conducted between 1982 and 2019 at 92 institutions from 25 countries. NAChRDB covers 41 methods and most annotations come from electrophysiology, mutagenesis, or ligand-binding assays, as well as from computational methods such as molecular docking and rate-equilibrium free-energy relationship analysis (Figure S1).

### Implementation

NAChRDB content is stored in the. json format, queried using Python, and rendered in the browser using Javascript and LiteMol, a WebGL-based technology for real-time in-browser rendering of large-scale macromolecular structures to ensure high responsiveness.^11^ NAChRDB is freely available online at https://crocodile.ncbr.muni.cz/Apps/NAChRDB/, and accessible through any modern internet browser on desktops and mobile devices. There is no need for installation or user registration. Interactive tutorials serve to help the user build progressively more complex searches and interpret the results.

**Figure 2:**
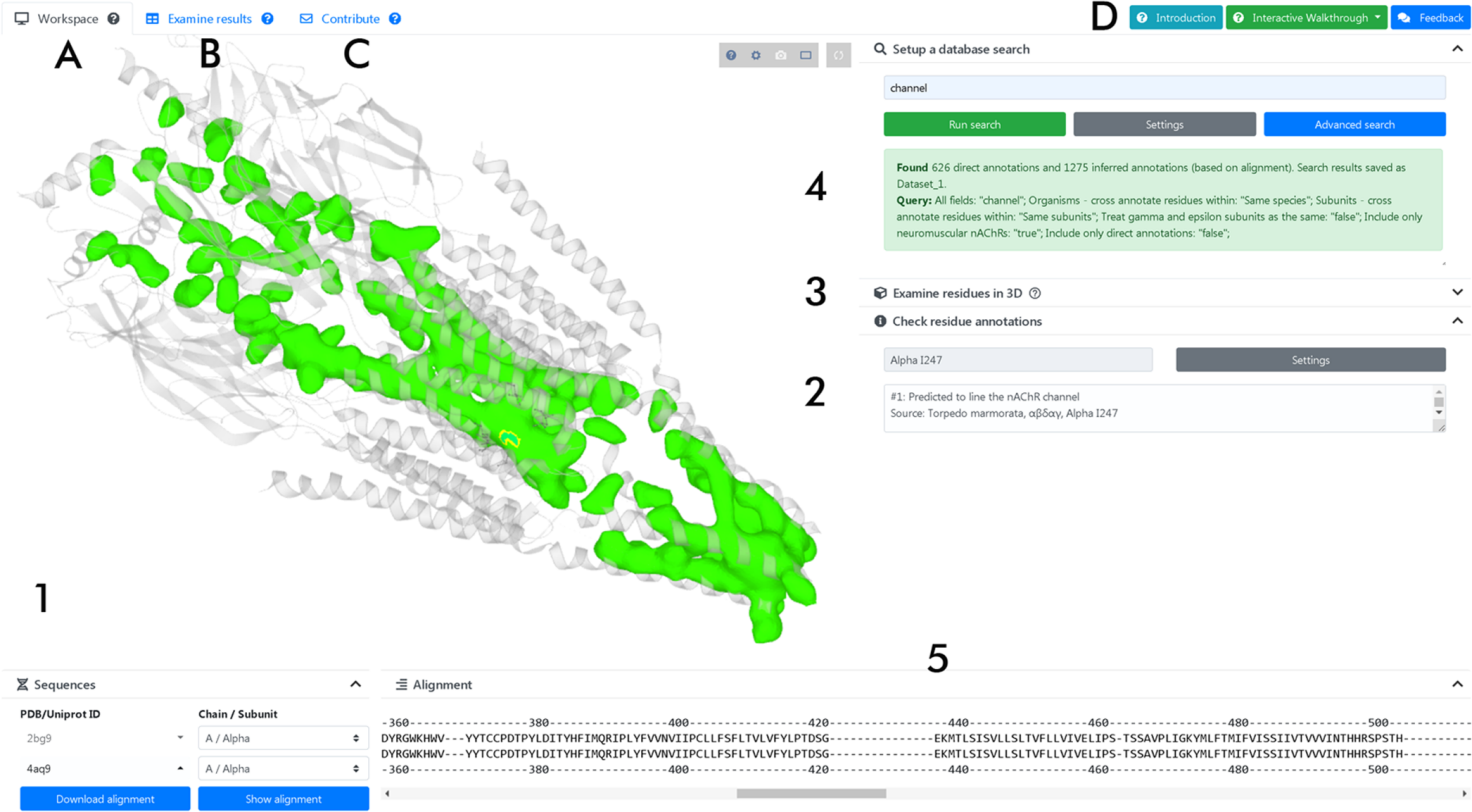
Recent screenshot of the NAChRDB workspace. The NAChRDB user interface is organized into tabs. The “Workspace” tab (A, active in the screenshot), which serves for conducting searches and examining models and sequences, contains: (1) a 3D viewer widget providing interactive visualization of nAChR 3D models, complete with (2) direct reporting of annotation records for a selected residue and (3) settings for adjusting the 3D representation (collapsed in the screenshot); (4) a search section providing extensive, PubMed-like search functionality for querying structure, function, and literature-related fields; and (5) a sequence alignment viewer is available for relevant comparisons and also allows listing any annotation records available for a selected residue. The “Examine results” tab (B) summarizes the search results in an interactive table that also allows users to filter data sets and perform additional operations. All results can be downloaded in the csv and json format for further processing. The “Contribute” tab (C) allows users to report annotations. A dedicated help section (D) provides access to key information about nAChRs and NAChRDB, together with interactive tutorials and case studies, as well as a user experience survey.

The NAChRDB interface (Figure 2) allows the users to search for residues that have specific annotations, as well as for annotations corresponding to specific residues. Beginners can run simple queries by typing a single keyword or selecting a single residue in the 3D model or amino acid sequence. Advanced users can set up complex searches using structure, function, and literature-related search fields and logical operators. Search results are saved as data sets, which can be analyzed directly inside the browser or downloaded in the. csv or. json format for further processing. Users may also seamlessly submit suggestions for annotations, thus contributing to maintaining NAChRDB up to date. These suggestions are manually curated before integration with NAChRDB.

### Example Use Cases

One click in the 3D visualizer or the amino acid sequence explorer immediately reveals whether any potential role has been reported for a specific residue, whereas a simple text search helps to quickly gauge the volume of knowledge associated with a specific topic. For example, a simple search using the keyword “agonist” quickly retrieves a substantial number of residues and annotations from studies focused on receptor–agonist interactions.

**Figure 3:**
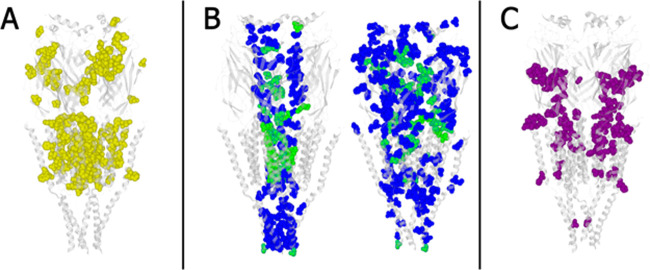
Case studies illustrating the usefulness of NAChRDB as a unified resource for structure–function annotations of neuromuscular nAChRs. Color coding on the 3D model: (A) yellow—residues discussed only in reports from nonhuman studies; (B) blue—residues predicted to affect the function based on channel-lining calculation (Pravda et al.^10^ and current study; left panel) or charge-profile analysis (Ionescu et al.^9^ and current study; right panel); green—residues predicted to affect the channel function based on channel-lining calculation (Pravda et al.^10^ and current study; left panel) or charge-profile analysis (Ionescu et al.^9^ and current study; right panel), but which were also studied elsewhere and reported to play a role (subset of blue); and (C) purple—residues for which some studies suggested an important role, whereas other studies suggested no important role. The 3D model of *Torpedo marmorata* neuromuscular nAChR (PDB ID: 2BG9)^12^ was used for graphical representation in all panels.

On the other hand, advanced searches can lead to important insights that can only be gained by examining individual findings in a larger context. For example, while much of the knowledge about NAChRs has been obtained via structural studies in *Torpedo* species and functional studies in murines, some residues thought to be critical for drug or agonist binding, channel gating, or *N*-glycosylation were never studied in human neuromuscular nAChR (Figure 3A). Thus, NAChRDB can be used to identify gaps in knowledge and design future investigations across different species. Furthermore, when conducting computational studies, NAChRDB can be used to put the new findings and subsequent predictions into context, as well as to adjust the study design toward confirmatory or exploratory analysis. For example, among the residues predicted to line the channel’s inner surface in *Torpedo* neuromuscular nAChR, only 28.5% have been studied to date and received annotations; similarly, among the residues predicted to contribute to protein-wide charge transfer networks that facilitate channel gating in *Torpedo* neuromuscular nAChR, only 18.5% have been studied to date and received annotations (Figure 3B). Finally, NAChRDB can be used to identify potential areas of ambiguity or controversy in the current knowledge. For example, much remains to be clarified regarding the relationship between channel opening kinetics and voltage dependency, especially in nAChRs where key ASP residues are mutated to noncharged residues (Figure 3C). The full case studies with step-by-step instructions on how to conduct the analysis are available on the NAChRDB web, whereas interesting residues highlighted by the analysis are included in the Supporting Information (Figure S2).

### Strengths and Limitations

NAChRDB goes beyond A7DB,^13^ in the direction of PDBe-KB,^14^ aiming to provide coherent access to structural, pharmacological, and physiological data for nAChRs, without limitation to a certain organism, tissue, type of receptor, or type of subunit. Most annotations currently stored in NAChRDB refer to neuromuscular nAChRs, but we are constantly updating the database and hope to increase coverage to all nAChRs and even nAChR-like proteins. As NAChRDB grows, we plan to integrate with PDBe-KB through FunPDBe to promote visibility and standardized access.

The relationship between publication and validation is central to the mission of NAChRDB, which is not to grant credibility to the reports, but rather to create a venue for assessing the credibility of reports in the proper context of the knowledge accumulated so far. In addition to annotations based on published studies, NAChRDB currently also contains annotations based on the findings of two computational investigations we performed in an effort to provide users with interesting opportunities for discussion and exploration. We hope that more researchers submit their findings to NAChRDB irrespective of the publication status because we firmly believe that the scientific community can only benefit from immediate access to information, as long as the publication status of the findings is clearly labeled. As such, every annotation record in NAChRDB contains either a DOI to the published source or a link to the source and the explicit mention “Unpublished as of...”, to ensure transparency.

NAChRDB is not simply a set of links to classifications, papers, or other databases, but a rich curated collection of relevant findings that describe structure–function relationships, uniquely suited for exploring the allosteric mechanisms underlying nAChR function and pathology. The relevant information is given directly in the search report, which is not only informative but also helps spot contradictory findings and inconsistent reporting. Many residues highlighted by state-of-the-art computational analyses have not been investigated to date, mainly because experimental studies are expensive and thus focus only on areas perceived to be of utmost importance. Furthermore, due to reporting bias, negative results are scarce. Thus, in addition to serving as an easy reference for structure–function information, we hope that NAChRDB will help promote the reporting of both positive and negative results, so that the scientific community may form a comprehensive picture of nAChR functioning. Currently, NAChRDB represents a powerful tool both in classrooms and in labs focused on the study of nAChRs. Ultimately, NAChRDB can also serve as a key starting point for unifying the state-of-art knowledge in the broad field of pentameric ligand-gated ion channels.

## Supporting Information Available

Coverage of NAChRDB, Supporting Information methods, and interesting examples (PDF)
3D model of the closed conformer of muscle nAChR used in the charge-profile analysis (PDB), 3D model of the open conformer of muscle nAChR used in the charge-profile analysis (PDB), values of atomic charges obtained using AtomicChargeCalculator with EEM and used in the charge-profile analysis (CSV), values of atomic charges obtained using NEEMP with EEM and used in the charge-profile analysis (CSV), and values of atomic charges obtained using NEEMP with QEq and used in the charge-profile analysis (CSV) (ZIP)
Scripts used to process the data included in NAChRDB (ZIP)

## Notes

NAChRDB is freely available via its dedicated server at https://crocodile.ncbr.muni.cz/Apps/NAChRDB/. All source data for NAChRDB were collected from publicly available databases and processed using publicly available tools or custom scripts (full details and scripts available in the Supporting Information). All data in NAChRDB are free to browse, process, or download via the NAChRDB web server, which also contains an interactive walkthrough for each case study described in this manuscript, as well as an interactive tutorial and plenty of tool tips. There is no login requirement or need for installation, and NAChRDB can be accessed via any modern internet browser on desktops or mobile devices.

## Dedication

This paper is dedicated to the memory of professor Jaroslav Koča—a scientist with a great vision, a wise mentor, and a warmhearted leader, who passed away while this paper was being peer-reviewed.
