# Supplementary material for: NAChRDB: A Web Resource of Structure–Function Annotations to Unravel the Allostery of Nicotinic Acetylcholine Receptors
Source: ACS Omega. 2021 Aug 31;6(36):23023–7. doi: 10.1021/acsomega.1c00817 (PMC8444218; doi:10.1021/acsomega.1c00817)
Supplement: Supplementary file 1 — ao1c00817_si_001.pdf [file ao1c00817_si_001.pdf]

NAChRDB: A Web Resource of Structure-Function Annotations to Unravel the Allostery of Nicotinic Acetylcholine Receptors

Aliaksei Chareshneu[1,2,‡,*], Purbaj Pant[1,2,‡], Ravi José Tristão Ramos[1,2], David Sehnal[1,2,3], Tuğrul Gökbel[4], Crina-Maria Ionescu[1,2], and Jaroslav Koča[1,2]

[1]CEITEC - Central European Institute of Technology, Masaryk University, Brno, Czech Republic

[2]National Centre for Biomolecular Research, Faculty of Science, Masaryk University, Brno, Czech Republic

[3]Protein Data Bank in Europe (PDBe), European Molecular Biology Laboratory, European Bioinformatics Institute (EMBL-EBI), Wellcome Genome Campus, Hinxton, UK

[4]Department of Molecular Biology and Genetics, Izmir Institute of Technology, İzmir, Turkey

[‡]These authors contributed equally to the work and should be considered co-first authors.

[*]To whom correspondence should be addressed.

ASSOCIATED CONTENT

The following files are available free of charge:

- Supporting Information: Coverage of NAChRDB, supplemental methods, interesting examples (PDF)
- File S1: 3D model of the closed conformer of muscle nAChR used in the charge-profile analysis (PDB)
- File S2: 3D model of the open conformer of muscle nAChR used in the charge-profile analysis (PDB)
- File S3: Values of atomic charges obtained using AtomicChargeCalculator with EEM and used in the charge-profile analysis (CSV)
- File S4: Values of atomic charges obtained using NEEMP with EEM and used in the charge-profile analysis (CSV)
- File S5: Values of atomic charges obtained using NEEMP with QEq and used in the charge-profile analysis (CSV)
- File S6: Scripts used to process the data included in NAChRDB (ZIP)

Coverage of NAChRDB

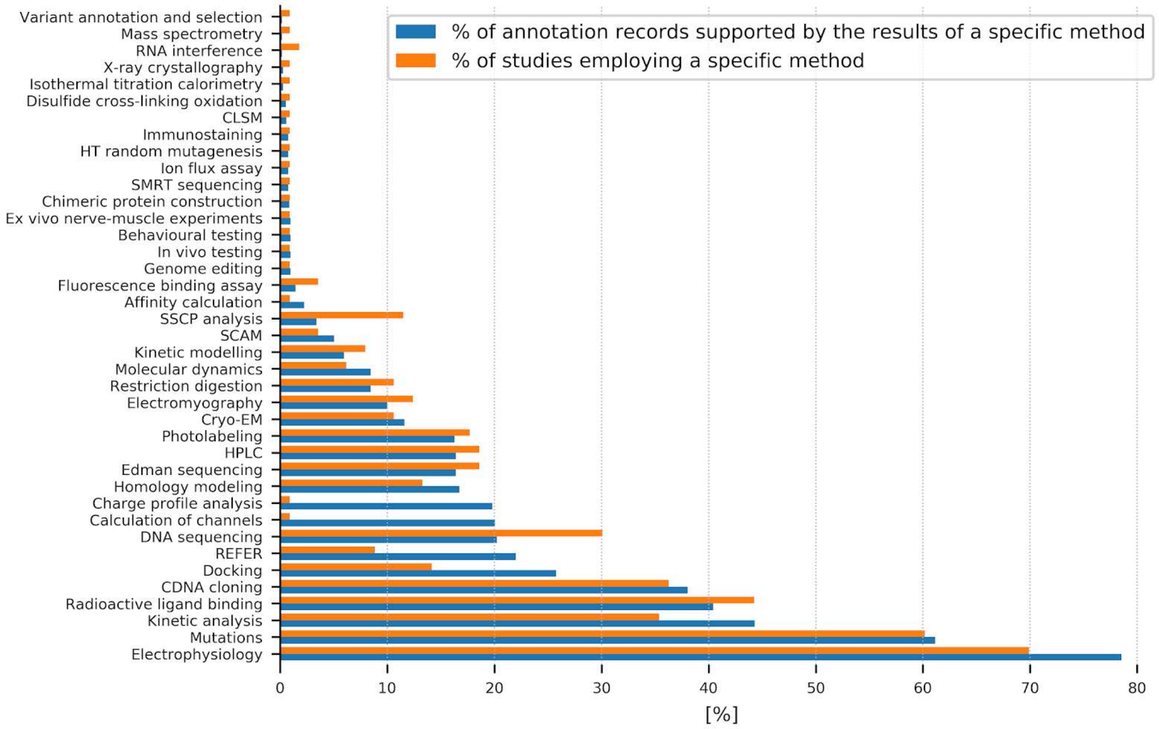

Figure S1. Coverage of NAChRDB. At the launch of NAChRDB, almost 70% of the studies included in the database employed electrophysiology, whereas electrophysiology findings supported about 80% of annotation records. Many studies employed multiple methods (e.g., electrophysiology and mutagenesis), and some annotation records are supported by evidence from multiple experiment types. The database is updated with curated annotations with every occasion. Abbreviations: CLSM, confocal laser scanning microscopy; HT random mutagenesis, high-throughput random mutagenesis; SMRT sequencing, single-molecule real-time sequencing; SSCP analysis, single-strand conformation polymorphism analysis; SCAM, substituted-cysteine accessibility method; HPLC, high-performance liquid chromatography; REFER, rate-equilibrium free-energy relationship.

Supplemental Methods

Retrieving PDB Models

On 23.08.2020, we searched the PDB in order to find all complete nAChR structures. The following search query was used:

(Full Text = ""nicotinic acetylcholine receptor"" OR Full Text = ""nicotinic receptor"" OR Full Text = ""acetylcholine receptor"") AND ((Structure Title CONTAINS PHRASE ""nicotinic acetylcholine receptor"" OR Structure Title CONTAINS PHRASE ""nicotinic receptor"" OR Structure Title CONTAINS PHRASE ""acetylcholine receptor"") AND Structure Title NOT CONTAINS WORDS ""muscarinic"" AND Source Organism Taxonomy Name = "Eukaryota" AND Source Organism Taxonomy Name NOT = "Mollusca" AND Total Number of Polymer Residues per Deposited Model >= 1500 AND Resolution <= 10).

After manual inspection, 11 PDB structures were added to NAChRDB (PDB IDs: 6UWZ, 6USF, 6UR8, 6PV7, 6PV8, 6CNJ, 6CNK, 5KXI, 4AQ9, 4AQ5, 2BG9).

Retrieving Amino Acid Sequences

On 30.04.2020, we searched UniProtKB to find complete nAChR sequences. The following search query was used:

*taxonomy:"Bilateria [33213]" "nicotinic acetylcholine receptor" NOT toxin length:[300 TO *] family:"ligand-gated ion channel tc 1 a 9 family acetylcholine receptor tc 1 a 9 1 subfamily" keyword:"Ion channel [KW-0407]" keyword:"Ligand-gated ion channel [KW-1071]" keyword:"Receptor [KW-0675]" keyword:"Ion transport [KW-0406]" keyword:"Transport [KW-0813]".*

The search retrieved 116 UniProt entries, of which 5 (UniProt IDs: Q9U298, Q60S81, P13908, P18257, P54247) were excluded because it was not possible to infer the subunit type according to commonly recognized typology (alpha, beta, delta, gamma, epsilon). Three UniProt entries corresponding to Torpedo marmorata nAChR subunits delta, beta, and gamma (UniProt IDs: Q6S3H8, Q6S3I0, Q6S3H9) had to be manually added to the list because they are not classified as members of the acetylcholine receptor (TC 1.A.9.1) subfamily in UniProtKB and were thus not retrieved by the automated search.

After manual inspection, data from 114 UniProt entries were included in NAChRDB (UniProt IDs: Q6S3H8, Q6S3I0, Q6S3H9, P43144, Q9JLB5, Q9UGM1, Q9GZZ6, P25162, P09478, P12390, P12392, P09484, P17644, P17787, P32297, P30926, P36544, P43681, P04757, P02708, Q9ERK7, P09483, Q8R4G9, O70174, P30532, Q8R493, P48182, Q04844, Q05941, Q9N587, Q23022, Q07001, Q15822, P48181, P09482, P22770, P20420, P11230, P04760, Q15825, Q05901, P49582, P04756, P12389, Q2MKA5, P25110, Q27218, P43143, P25108, P02716, P09660, Q9R0W9, P12391, P07510, P20782, P09690, P25109, P02709, Q98880, P18916, P54131, Q866A2, P48180, P04755, Q8BMN3, P04758, P09481, P54244, P09479, P26153, P13536, P02712, Q93149, P04759, G5EG88, P26152, P02715, P09480, Q07263, P02713, P02717, P43679, Q91X60, Q9PTS8, Q5IS77, P49581, P02718, P02714, P02710, Q9I8C7, Q5IS76, Q8SPU7, P18845, Q8SPU6, P19370, O76554, P02711, Q5IS52, Q5IS75, Q5IS51, Q19AE6, P45963, P09628, P22456, P05376, P54246, P05377, P49580, P49579, A8WQK3, P23414, Q8JFN7, Q68RJ7, P91766).

Charge-Profile Analysis

Residues potentially involved in charge transfer networks facilitating channel gating were predicted using a modified charge profile analysis[1]. Briefly, the following steps were employed: (i) conformers were generated based on normal mode analysis; atomic charges for each conformer were computed using three conformationally-dependent methods; residue charges were then compared across conformers; outliers were reported based on robust statistical analysis.

The starting molecular structures of muscle nAChR were obtained from PDB entries 4AQ5 and 4AQ9[2]. The TRITON software[3] was used to protonate both structures and correct the known minor structural issues. The web server *elNémo*[4], with default settings, was used to calculate the low-frequency normal modes and generate normal-mode-perturbed models of muscle nAChR. In *elNémo*, normal-mode calculation is based on the harmonic approximation of the potential energy function around a minimum energy conformation, where a single-parameter Hookean potential is used. The Elastic Network Model[4,5] is then applied to generate models that are perturbed with a given amplitude in the direction of a single normal mode. In our study, a total of 11 low-energy models of each of the two muscle nAChR structures (PDB entries 4AQ5 and 4AQ9) were generated, perturbed in the direction of the 5 lowest-frequency normal modes (parameters: minimum perturbation, -100; maximum perturbation, 100; step size, 20; and cutoff for the elastic interaction, 8 Å). We then chose for further analysis the two most distinct models (highest RMSD with respect to C-α atoms). The channel radius profiles were computed for these two models using the web server ChExVis[6], with default settings. The model with a wider channel profile in the known gating area was designated as the open conformer, and the other model was designated as the closed conformer. The 3D models of the closed and open conformers of muscle nAChR used to derive the results included in nAChRDB are available in .pdb format (Supporting Information: files S1.pdb and S2.pdb).

The protonated structures were carefully inspected in PyMol v2.2.0.0[7]. To ensure statistical robustness, atomic charges were computed using several methods based on the concepts of electronegativity equalization[8] or charge equilibration[9]. The software AtomicChargeCalculator[10] was used with the following setup: total charge for each conformer, -16 e; parameter set, EX-NPA 6-31Gd PCM[11]; computation method, Full EEM. Additionally, the software NEEMP[12] was used with the following setup: EEM[13] and QEq[12,13].

These methods compute atomic charges that respond to changes in molecular conformation. Therefore, despite having the same total charge and using the same computation setup, the closed and open conformers of nAChR exhibited different atomic charges. The values of the atomic charges used to derive the results included in NAChRDB are available in .csv format (Supporting Information: files S3.csv, S4.csv, S5.csv).

For each amino acid residue r, the total charge Q_r was obtained as the algebraic sum of the atomic charges qi on all the atoms that belong to residue r:

(1) $Q_r = \sum_{i=1}^{n_r} qi$

where n_r is the number of atoms in residue r. The significance of residue r as a contributor to the charge transfer network was evaluated based on the absolute difference D_r between its charge in the open conformer Q_{ro}, and its charge in the closed conformer Q_{rc}:

(2) $D_r = \left| Q_{ro} - Q_{rc} \right|$

Residue r was considered significant if its D_r lay above the upper inner fence of the distribution of absolute differences for the entire molecule:

(3) $D_r \geq D^{75} + 1.5 \cdot \left(D^{75} - D^{25} \right)$

where D^{75} and D^{25} represent the upper and lower quartiles, respectively, of the distribution of D_r values for all residues in muscle nAChR. This significance threshold can be further extended to:

(4) $D_r \geq D_{ave} + 2 \cdot \sigma$

where D_{ave} and σ represent the average value and the standard deviation, respectively, of the distribution of D_r values for all residues in muscle nAChR.

Identification of Residues Lining the Channel

The list of channel-lining residues for PDB entries included in NAChRDB was obtained from ChannelsDB[14] using a suitable API request (only PDB entries covered by ChannelsDB): https://webchem.ncbr.muni.cz/API/ChannelsDB/PDB/pdb_id, where pdb_id is the PDB ID.

Sequence Alignment

All original annotations are curated manually and mapped onto homologous residues using sequence alignment. Multiple sequence alignment of all protein sequences included in NAChRDB was performed using Clustal W with default settings[15]. The alignment was inspected manually upon completion. All relevant alignments (within the same subunit) are available for download from the NAChRDB web.

Interesting Examples

We performed several case studies to showcase the usefulness of NAChRDB. These case studies are available on the NAChRDB web, together with fully interactive tutorials and explanations, as well as access to all data. Here we would like to highlight a few interesting examples that can easily serve as a starting point for classroom discussions and may even spark new investigations.

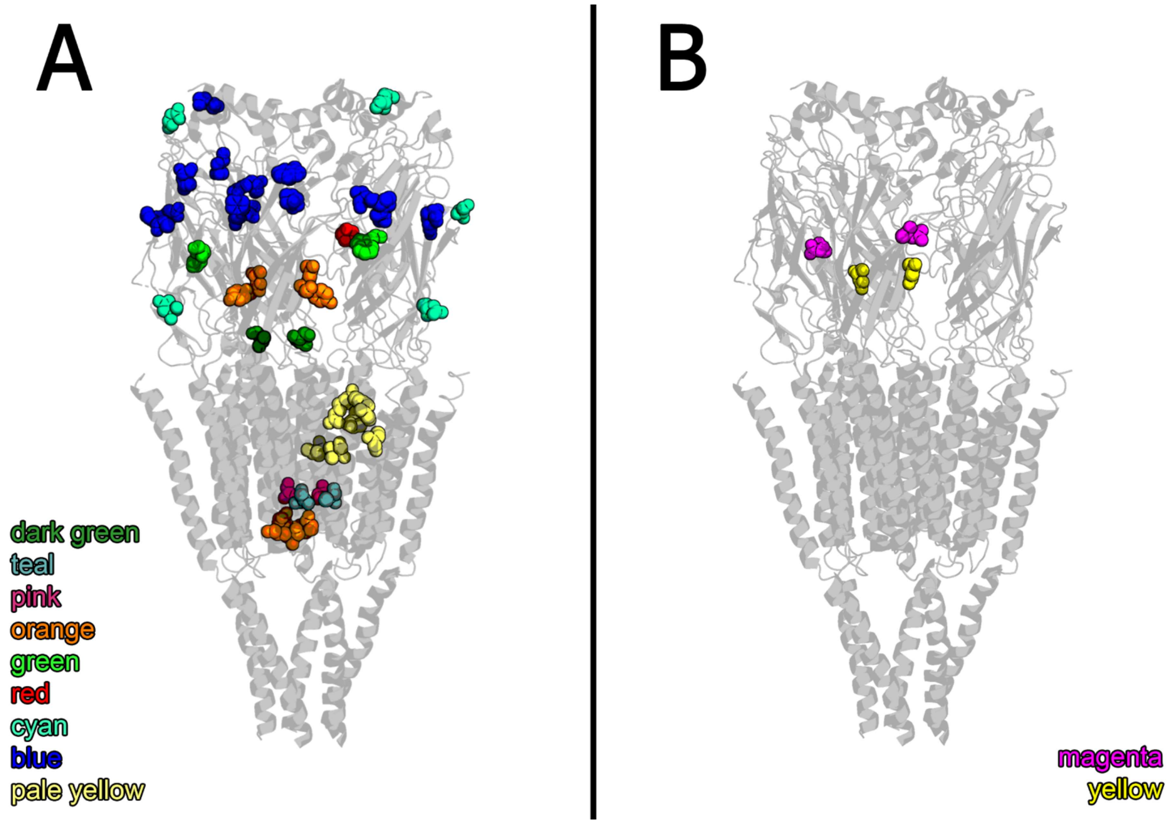

Figure S2. Interesting residues highlighted by the analyses of case studies. The 3D model of *T. marmorata* nAChR (PDB ID: 4AQ9)[2] was used. The residues are represented as overlapping Van der Waals spheres and color-coded according to their reported role. The residue labels include the subunit(s), residue name, and residue number, which is given according to the neuromuscular nAChR sequence from the respective organism or inferred based on alignment.

(A) Selection of functionally important residues not yet studied in humans. Color coding:

- dark green – αγVAL46 and αδVAL46 were suggested to serve as a key structural link between the extracellular and transmembrane domains of the neuromuscular nAChR in *T. marmorata*[16];
- teal and pink – αγSER248, αδSER248, βSER254, and δSER262 form a binding site for a channel-blocking non-competitive antagonist of the neuromuscular nAChR in *T. marmorata*[17];
- orange – αγTYR127, αδTYR127, αγASP97, αδASP97, αγTHR244, αδTHR244, αγLYS242, and αδLYS242 participate in the channel gating process of neuromuscular nAChR in murines[18,19];
- green – mutations of γTRP55 and δTRP57 change the agonist affinity of neuromuscular nAChR in murines[20];
- red – mutations of δPRO123 disable one ACh binding site in murine neuromuscular nAChR[21];
- cyan – γASN68, γASN141, δASN70, δASN143, and δASN208 may form N-glycosylation sites in the neuromuscular nAChR of *T. californica*[22,23];
- blue – αγASP195, αδASP195, γLEU77, αγPRO194, αδPRO194, αγCYS193, αδCYS193, γLYS10, αγTRP86, αδTRP86, αγPRO88, αδPRO88, γTYR104, γILE80, and δTYR212 may contribute to physostigmine binding sites in the neuromuscular nAChR of *T. californica*[24];
- pale yellow and pink - δTHR274, δPHE232, δCYS236, δARG277, αγSER248, αδSER248, βSER254, βVAL261, and δVAL269 may form propofol binding sites in the neuromuscular nAChR of *T. californica*[25].

(B) Selection of residues for which contradictory findings have been reported:

- Mutation of αASP200 (magenta) to GLN in the mouse neuromuscular nAChR expressed in human kidney cells was reported to have a dramatic effect on channel opening rate[26] but a non-significant effect on slope conductance or voltage dependency[27].

- Some mutations of αASP97 (yellow) in mouse neuromuscular nAChR were shown to dramatically change gating kinetics[18,28], whereas other mutations were found to have only a marginal effect[29].

References

(1) Ionescu, C. M.; Svobodová Vařeková, R.; Prehn, J. H. M.; Huber, H. J.; Koča, J. Charge profile analysis reveals that activation of pro-apoptotic regulators bax and bak relies on charge transfer mediated allosteric regulation. *PLoS Comput. Biol.* **2012**, DOI: 10.1371/journal.pcbi.1002565.

(2) Unwin, N.; Fujiyoshi, Y. Gating movement of acetylcholine receptor caught by plunge-freezing. *J. Mol. Biol.* **2012**, DOI: 10.1016/j.jmb.2012.07.010.

(3) Prokop, M.; Damborsky, J.; Koca, J. TRITON: In silico construction of protein mutants and prediction of their activities. *Bioinformatics* **2000**, *16*, 845–846.

(4) Suhre, K.; Sanejouand, Y.-H. ElNemo: a normal mode web server for protein movement analysis and the generation of templates for molecular replacement. *Nucleic Acids Res.* **2004**, *32*, W610-614.

(5) Tama, F.; Sanejouand, Y. H. Conformational change of proteins arising from normal mode calculations. *Protein Eng.* **2001**, *14*, 1–6.

(6) Masood, T. Bin; Sandhya, S.; Chandra, N.; Natarajan, V. CHEXVIS: A tool for molecular channel extraction and visualization. *BMC Bioinf.* **2015**, DOI: 10.1186/s12859-015-0545-9.

(7) *The PyMOL Molecular Graphics System, Version 2.2.0.0*; Schrödinger, LLC: New York, 2018. https://github.com/schrodinger/pymol-open-source/releases (accessed 2018-09-30).

(8) Mortier, W. J.; Ghosh, S. K.; Shankar, S. Electronegativity-equalization method for the calculation of atomic charges in molecules. *J. Am. Chem. Soc.* **1986**, *108*, 4315–4320.

(9) Rappe, A. K.; Goddard, W. A. Charge equilibration for molecular dynamics simulations. *J. Phys. Chem.* **1991**, *95*, 3358–3363.

(10) Ionescu, C. M.; Sehnal, D.; Falginella, F. L.; Pant, P.; Pravda, L.; Bouchal, T.; Svobodová Vařeková, R.; Geidl, S.; Koča, J. AtomicChargeCalculator: Interactive web-based calculation of atomic charges in large biomolecular complexes and drug-like molecules. *J. Cheminf.* **2015**, DOI: 10.1186/s13321-015-0099-x.

(11) Ionescu, C.-M.; Geidl, S.; Svobodová Vařeková, R.; Koča, J. Rapid Calculation of Accurate Atomic Charges for Proteins via the Electronegativity Equalization Method. *J. Chem. Inf. Model.* **2013**, DOI: 10.1021/ci400448n.

(12) Raček, T.; Pazúriková, J.; Svobodová Vařekova, R.; Geidl, S.; Křenek, A.; Falginella, F. L.; Horský, V.; Hejret, V.; Koča, J. NEEMP: Software for validation, accurate calculation and fast parameterization of EEM charges. *J. Cheminf.* **2016**, DOI: 10.1186/s13321-016-0171-1.

(13) Raček, T.; Schindler, O.; Svobodová Vařeková, R.; Koča, J. Empirical methods for calculation of partial atomic charges – applicability for proteins? In *ENBIK2018, ENBIK2018 conference proceedings*, Prague, Czech Republic, June 11−13, 2018; Čech, P., Svozil, D., Eds.; University of Chemistry and Technology, Prague: Prague, Czech Republic, 2018; p. 73. ISBN 978-80-7592-017-1

(14) Pravda, L.; Sehnal, D.; Svobodová Vařeková, R.; Navrátilová, V.; Toušek, D.; Berka, K.; Otyepka, M.; Koča, J. ChannelsDB: database of biomacromolecular tunnels and pores. *Nucleic Acids Res.* **2018**, DOI: 10.1093/nar/gkx868.

(15) Thompson, J. D.; Higgins, D. G.; Gibson, T. J. CLUSTAL W: Improving the sensitivity of progressive multiple sequence alignment through sequence weighting, position-specific gap penalties and weight matrix choice. *Nucleic Acids Res.* **1994**, *22*, 4673–4680.

(16) Miyazawa, A.; Fujiyoshi, Y.; Unwin, N. Structure and gating mechanism of the acetylcholine receptor pore. *Nature* **2003**, *423*, 949–955.

(17) Hucho, F.; Oberthür, W.; Lottspeich, F. The ion channel of the nicotinic acetylcholine receptor is formed by the homologous helices M II of the receptor subunits. *FEBS Lett.* **1986**, *205*, 137–142.

(18) Purohit, P.; Auerbach, A. Acetylcholine receptor gating: Movement in the α-subunit extracellular domain. *J. Gen. Physiol.* **2007**, *130*, 569–579.

(19) Zhang, H.; Karlin, A. Contribution of the beta subunit M2 segment to the ion-conducting pathway of the acetylcholine receptor. *Biochemistry* **1998**, DOI: 10.1021/bi980143m.

(20) Nayak, T. K.; Chakraborty, S.; Zheng, W.; Auerbach, A. Structural correlates of affinity in fetal versus adult endplate nicotinic receptors. *Nat. Commun.* **2016**, DOI: 10.1038/ncomms11352.

(21) Gupta, S.; Purohit, P.; Auerbach, A. Function of interfacial prolines at the transmitter-binding sites of the neuromuscular acetylcholine receptor. *J. Biol. Chem.* **2013**, DOI: 10.1074/jbc.M112.443911.

(22) Chiara, D. C.; Cohen, J. B. Identification of amino acids contributing to high and low affinity d-tubocurarine sites in the Torpedo nicotinic acetylcholine receptor. *J. Biol. Chem.* **1997**, *272*, 32940–32950..

(23) Strecker, A.; Franke, P.; Weise, C.; Hucho, F. All potential glycosylation sites of the nicotinic acetylcholine receptor δ subunit from Torpedo californica are utilized. *Eur. J. Biochem.* **1994**, *220*, 1005–1011.

(24) Hamouda, A. K.; Kimm, T.; Cohen, J. B. Physostigmine and galanthamine bind in the presence of agonist at the canonical and noncanonical subunit interfaces of a nicotinic acetylcholine receptor. *J. Neurosci.* **2013**, *33*, 485–494.

(25) Jayakar, S. S.; Dailey, W. P.; Eckenhoff, R. G.; Cohen, J. B. Identification of propofol binding sites in a nicotinic acetylcholine receptor with a photoreactive propofol analog. *J. Biol. Chem.* **2013**, DOI: 10.1074/jbc.M112.435909.

(26) Akk, G.; Sine, S.; Auerbach, A. Binding sites contribute unequally to the gating of mouse nicotinic alpha D200N acetylcholine receptors. *J. Physiol. (Oxford, U. K.)* **1996**, *496 (Pt 1)*, 185–196.

(27) Dworakowska, B.; Nurowska, E.; Dołowy, K. Hydrocortisone inhibition of wild-type and αD200Q nicotinic acetylcholine receptors. *Chem. Biol. Drug Des.* **2018**, DOI: 10.1111/cbdd.13325.

(28) Chakrapani, S.; Auerbach, A. A speed limit for conformational change of an allosteric membrane protein. *Proc. Natl. Acad. Sci. U. S. A.* **2005**, *102*, 87–92.

(29) Chakrapani, S.; Bailey, T. D.; Auerbach, A. The Role of Loop 5 in Acetylcholine Receptor Channel Gating. *J. Gen. Physiol.* **2003**, *122*, 521–539.
