# Supplementary material for: NAChRDB: A Web Resource of Structure–Function Annotations to Unravel the Allostery of Nicotinic Acetylcholine Receptors
Source: ACS Omega. 2021 Aug 31;6(36):23023–7. doi: 10.1021/acsomega.1c00817 (PMC8444218; doi:10.1021/acsomega.1c00817)
Supplement: Supplementary file 3 — ao1c00817_si_003.zip [file ao1c00817_si_003.zip › NAChRDB_scripts/README_NAChRDB.pdf]

Key NAChRDB scripts

nachrdb/builder.py

Builds the database files.

Required input

- PDB files in `.pdb` or `.ent` formats placed in `./db_build/pdb_src/` (for constructing PDB-based entries of the database)
- `./db_build/literature_src/Literature.csv` with bibliographic data
- UniProt XML files placed in `./db_build/uniprot_src/` (for constructing UniProt-based entries)
- `./db_build/annotation_src/Annotations_modified.csv` with annotation data from the literature
- `./db_build/annotation_src/Charge_analysis_annotations.csv` with annotation data obtained using charge-profile analysis

Main output

- Files with sequence alignments grouped by subunit type in `./db_build/alignment_src/clustalw2/`
- Files with amino acid sequences of individual chains/subunits in `./nachrdb/sequences_by_chains/`
- JSON file used to store the data in the NAChRDB (`./nachrdb/built_nAChR_Db_00001_v0.1.0.json`)
- JSON files with exact positions of amino acid residues in a sequence for each chain/subunit in `./nachrdb/exact_residue_positions/` (used to handle chain breaks)
- A file with the list of methods employed in studies which are included in NAChRDB (`./nachrdb/methods_list.txt`)
- Files with annotations for each chain/subunit used in frontend part in `./nachrdb/Litemol_Annotations/`
- A file listing individual chains/subunits IDs (PDB or UniProt ID + chain/subunit ID) in NAChRDB along with the organism names they originate from, and plain English name of subunit (`./nachrdb/chain_to_subunit.txt`)
- A file with the numbers of PDB- and UniProt-based entries in the NAChRDB (`./nachrdb/nachrDBnumbers.txt`)

Usage

From the root directory:

```
cd nachrdb
python3 builder.py > log.txt
```

NOTE:
It is recommended to redirect the output to another file as in the example

nachrdb/json-php-recoding.php

Processes the JSON file used to store the data in the NAChRDB to identify and/or fix potential formatting issues.

Required input

- JSON file used to store the data in the NAChRDB (`./nachrdb/built_nAChR_Db_00001_v0.1.0.json`)

Main output

- JSON file used to store the data in the NAChRDB (`./nachrdb/dbBuild.json`).

Usage

From the root directory:

```
cd nachrdb
php json-php-recoding.php
```

alignments_for_frontend/seq_aln_script.py

Performs amino acid sequence aligment of chains/subunits in NAChRDB, splits the alignment output onto files corresponding to the individual chains/subunits for displaying in web browser.

Required input

- Files with amino acid sequences of individual chains/subunits in `./nachrdb/sequences_by_chains/` (generated by nachrdb/builder.py)

Main output

- FASTA files with parts of sequence alignment corresponding to individual chains/subunits (in `./alignments_for_frontend/ind_chain_aln_output/`)
- Prints to stdout generated HTML code that is used in dropdown menus in frontend part.

Usage

From the root directory:

```
cd alignments_for_frontend
python3 seq_aln_script.py
```

remove_last_output.sh

Removes the output files of the last run of other NAChRDB scripts and **pycache** folders

Required input

- N/A

Main output

- N/A

Usage

From the root directory:

```
bash remove_last_output.sh
```
